# The Clinical Next‐Generation Sequencing Database: A Tool for the Unified Management of Clinical Information and Genetic Variants to Accelerate Variant Pathogenicity Classification

**DOI:** 10.1002/humu.23160

**Published:** 2017-01-11

**Authors:** Shin‐ya Nishio, Shin‐ichi Usami

**Affiliations:** ^1^ Department of OtorhinolaryngologyShinshu University School of MedicineMatsumoto CityJapan

**Keywords:** database, variant, clinical sequence, next‐generation sequencing

## Abstract

Recent advances in next‐generation sequencing (NGS) have given rise to new challenges due to the difficulties in variant pathogenicity interpretation and large dataset management, including many kinds of public population databases as well as public or commercial disease‐specific databases. Here, we report a new database development tool, named the “Clinical NGS Database,” for improving clinical NGS workflow through the unified management of variant information and clinical information. This database software offers a two‐feature approach to variant pathogenicity classification. The first of these approaches is a phenotype similarity‐based approach. This database allows the easy comparison of the detailed phenotype of each patient with the average phenotype of the same gene mutation at the variant or gene level. It is also possible to browse patients with the same gene mutation quickly. The other approach is a statistical approach to variant pathogenicity classification based on the use of the odds ratio for comparisons between the case and the control for each inheritance mode (families with apparently autosomal dominant inheritance vs. control, and families with apparently autosomal recessive inheritance vs. control). A number of case studies are also presented to illustrate the utility of this database.

## Introduction

Recent advances in molecular genetic analysis, particularly in analyses using next‐generation sequencing (NGS), have drastically accelerated the identification of novel genes involved in many inherited diseases and expanded the disease phenotype spectrum of known disease‐causing genes [Ng et al., 2010; Ku et al., 2011; Rabbani et al., 2012; Katsanis and Katsanis, 2013]. These new technologies have led to significant breakthroughs in the field of human genetics research; however, at the same time, they have also given rise to new challenges in the interpretation of the pathogenicity of an extraordinary number of newly identified genomic variants.

Recently, new guidelines for sequence variant interpretation have been published by the American College of Medical Genetics and Genomics (ACMG) and the Association for Molecular Pathology (AMP) [Richards et al., 2015]. According to these guidelines, variant pathogenicity should be interpreted by gathering evidence from various sources, such as the position and type of variant, the results of family segregation analysis, the survey results of many kinds of database including control populations as well as disease‐specific databases, the results of in vitro experiments, and the results of in silico prediction programs. This approach to variant classification was appropriately designed for use in a clinical diagnostic setting; however, this approach required considerable time and effort to assess all the updated information from many data sources as well as in‐house variant information. These guidelines also employed a statistical approach to the classification of variant pathogenicity. The prevalence of variants in affected individuals is significantly higher than that in the control population (over fivefold based on the odds ratio obtained from the case vs. control) and the 95% confidence interval around the estimated of odds ratio does not include 1.0. This was regarded as evidence supporting the pathogenicity of the variant.

In this report, we describe an original database software package, the Clinical Next‐Generation Sequencing Database (Clinical NGS DB), which can be utilized for efficient clinical NGS analysis of inherited diseases through the collection of data for a large number of variants as well as clinical information in a unified interface.

This database software offers a two‐featured approach to variant pathogenicity classification. The first of these approaches is a phenotype similarity‐based approach. In many diseases, a phenotype–genotype or gene‐to‐phenotype correlation has been reported [Bork et al., 2001; Astuto et al., 2002; Tsukada et al., 2010], and this correlation provides important information for variant classification. In this database, it is easy to compare the detailed phenotype of each patient to the averaged phenotype with the same gene mutation at the variant or gene level. It is also possible to browse patients with the same gene mutation quickly and compare many patient phenotypes with each other. This information is useful for the interpretation of variant pathogenicity.

The other approach is a statistical approach to variant pathogenicity classification that uses the odds ratio for comparisons between the case and the control in each inheritance mode (families with apparently autosomal dominant inheritance vs. control, and families with apparently autosomal recessive inheritance vs. control). Most of the pathogenic variants for autosomal recessive inherited disorders are preferentially observed among families with apparently autosomal recessive inheritance or sporadic cases and are seldom identified among families with apparently autosomal dominant inheritance. The occurrence of these variants identified in families with apparently autosomal dominant inheritance was considered to show the same prevalence as the carrier frequencies in the control population. In contrast, pathogenic variants for autosomal dominant inherited diseases should be mainly identified among families with apparently autosomal dominant inheritance and not usually identified from families with apparently autosomal recessive inheritance or sporadic cases. The occurrence of an autosomal dominant inherited pathogenic variant among families with apparently autosomal recessive inheritance or sporadic cases was considered a de novo mutation or the incomplete penetrance of the variant. The effectiveness of variant classification by both approaches is expected to increase as more data are accumulated.

In addition to these features, this database software is able to manage public control population allele frequency information, disease‐specific database annotation information, in silico predictions. And, by thorough use of these data, this database software automatically filter the identified variants. This software is, therefore, able to improve the efficiency of variant classification and clinical genetic diagnosis of inherited diseases using relatively few computer resources.

## Implementation and Overview

### Clinical NGS DB Workflow

**Figure 1:**
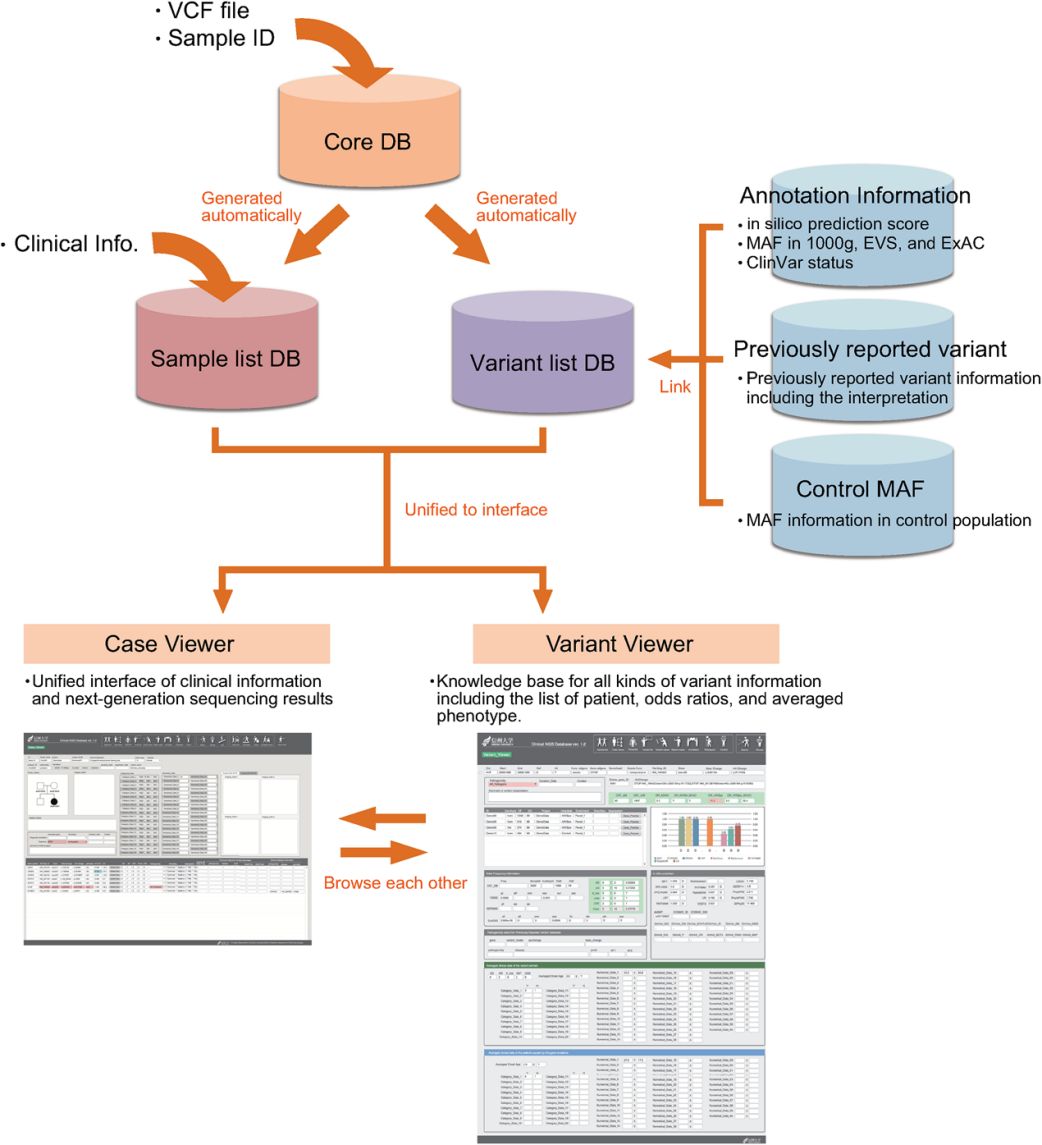
Overview of the Clinical NGS database. This database software was developed for the unified management of the detailed clinical information of each patient and next‐generation sequencing analysis results. This database was also intended to assist in efficient variant pathogenicity interpretation by gathering all knowledge about the variant and comparing the clinical features of each patient. Inheritance mode‐specific odds ratios, calculated automatically, are also useful for variant interpretation.

An overview of the Clinical NGS DB is shown in Figure 1. This database software is focused on NGS data management and clinical diagnosis using the patient's clinical information, thus the data analysis pipeline from mapping to the variant call is not included in this software.

VCF files (according to the VCF ver. 4.1 format) are a commonly used file format for sequence variant information that was originally developed by the 1000 Genomes Project [1000 Genomes Project Consortium et al., 2012; http://www.1000genomes.org/wiki/Analysis/vcf4.0]. Many types of analysis software, including sequencer developer‐provided software (MiSeq reporter, Torrent Suit software), enrichment kit provider software (SureCall), commercial analyzing software (Strand NGS, CLC genomic workbench), as well as many types of free software (samtools, GATK unified genotyper, or haplotype caller) support this format.

The Clinical NGS DB supports VCF files, so it is compatible with any sequencer platform, capture kit, or analysis pipeline.

As a first step, VCF files are imported into the database with the patient ID, sequencing platform information, and target panel information. After VCF file incorporation, the database software generates a sample list and identified variant list semi‐automatically (simply click on the “database update” icon). By using the variant list generated from the VCF files, a tab‐separated format text file is exported for annotation. This variant list file is formatted in the ANNOVAR variant description format. Based on this variant list, annotation information generated by the wANNOVAR or the ANNOVAR software [Wang K et al., 2010; Chang and Wang, 2012] is imported into the database. This annotation information is used for variant filtering for each sample.

The sample list automatic generated from the VCF files is able to manage the detailed clinical information of each patient. This version of the software is able to manage nine sets of graphical data, 40 sets of numerical data, and 20 sets of categorical data for each patient. These clinical data are used for calculating the averaged phenotype at each variant or gene level. This software is also able to manage the results of other genetic analyses, such as Sanger sequencing and TaqMan genotyping, as an option.

This database software has two main user interfaces; the “Case Viewer” and “Variant Viewer.” The “Case Viewer” is an interface allowing efficient clinical sequencing for the diagnosis of each patient (Supp. Fig. S1). This interface allows the user to access all the patient clinical information including Sample ID, Project Name, Pedigree, and variant information after automatic filtering including “protein affecting variants,” “low minor allele frequency or absent among control population,” “previously reported pathogenic variants,” and so on. This interface is also useful for managing “direct sequence conformation results,” “family segregation results,” and “diagnosis.” The “Variant Viewer” is an interface allowing the efficient assessment of pathogenicity for each variant (Supp. Fig. S2). In this interface, you can browse the whole variant information such as the patient IDs of those carrying the same variant and annotation information including computer prediction score and minor allele frequency (MAF) information from the 1000 Genomes Project [1000 Genomes Project Consortium et al., 2012], EVS6500 [Fu W et al., 2013], ExAC (http://exac.broadinstitute.org.], ClinVar [Landrum et al., 2014], and other databases. This interface also provides averaged clinical information and the standard deviation of the patients carrying the same variant for the easy comparison of patient phenotypes.

### Software and Hardware

The Clinical NGS DB is built on FileMaker Pro ver. 12 to facilitate easy start up and easy maintenance for non‐PC specialists such as wet lab researchers or clinicians. It can easily be used to build its own database on either a Windows or Macintosh platform with FileMaker Pro software (FileMaker Inc., Santa Clara, CA). The network sharing option of FileMaker Pro software makes it possible for up to five users to access the database at the same time. If access to a Web‐based server (to enable access to the database via a general Web‐browser without the need for software installation on the client computer) by six or more users at the same time is required, FileMaker server software offers a good solution. For more information, please refer to the FileMaker Inc. website (http://www.filemaker.com).

We recommend the following hardware specifications for running the software on the server.

Processor: Intel ® Core i5 1.6 GHz or faster
RAM: 4 GB or higher
Hard drive: 8GB or more space
LAN: Gigabit Eathernet
Software Requirement: FileMaker Pro ver. 12 or later

We confirmed that this database software can run comfortably on MacBook Air with a 1.6 GHz Intel Core i5 processor, 8 GB memory, and 256 GB Solid State Drive (SSD) in cases with target re‐sequencing data for 2,000 or more patients.

The maximum file size for one database was about 8 TB, which corresponds to the re‐sequencing data for about 8 million patients.

### Patient Consent and Study Approval

Informed written consent was obtained from all subjects. This study was approved by the Shinshu University Ethical Committee as well as the ethical committees of each of the other participating institutions described previously [Nishio and Usami, 2015].

## Results and Discussion

Using the Clinical NGS DB software, we developed a Japanese deafness variation database including a total of 4,052 target re‐sequencing analysis results for 68 known deafness genes. A total of 3,719 of the 4,052 samples were results for Japanese hearing loss patients (including 832 families with apparently autosomal dominant or mitochondrial inheritance, 2,370 families with apparently autosomal recessive inheritance or sporadic cases and 517 cases with unknown family history) and 333 were for in‐house Japanese controls.

From these samples, we identified 26,073 unique variants (a total of 1,497,366 variants were detected in the 4,052 samples). Among the 26,073 unique variants, 31.6% (8,232) were located in the exonic region, 1.5% (401) were located in the exonic splicing junction region, 46.9% (12,237) were located in intronic region, and 1.3% (345) were located in splicing junctions. The others were located in the 3′‐untranslated region (UTR), 5′‐UTR, non‐coding RNA, or intergenic regions. Among the exonic region variants, 72.7% (5,986 out of 8,232) affected proteins (4,806 missense variants, 166 nonsense variants, 334 frame shift deletions, 245 frame shift insertions, 129 frame shift multi‐base substitutions, 88 non‐frame shift deletions, 32 non‐frame shift insertions, 173 non‐frame shift multi‐base substitutions, and eight stop loss mutations).

We filtered these variants using the Clinical NGS DB filtering function as follows. (1) Variants previously reported as “pathogenic” or “likely pathogenic” in the ClinVar [Landrum et al., 2014] or Deafness Variation Database [Shearer et al., 2014] were not filtered, regardless of the MAF or other criteria. (2) Variants with a MAF >1% in the 1000 Genomes Project, the exome variant server, the exome aggregation consortium, and the human genetic variation database, which contains 1,200 Japanese exome data [Higasa et al., 2016], 2KJPN containing 2,000 Japanese control genome data [Nagasaki et al., 2015], and the 269 in‐house Japanese normal hearing controls, were filtered. (3) Intronic and synonymous variants with a scSNV [Jian et al., 2014] splicing score of less than 0.6 were filtered out. (4) Variants only identified in the controls were removed. Finally, 4,253 variants remained as candidates for further analysis.

The filtering parameters of the Clinical NGS DB can be modified if necessary. The variant filtering procedure used here is just an example, and the filtering parameters should be modified to the appropriate values for each disorder. In cases where no diagnostic candidates were identified by the standard filtering procedure, we should evaluate the individual filtered variants. The odds ratio provides a powerful tool for such variant re‐assessment (e.g., variants in the intronic or intergenic regions that are found only in the patients are good candidates for further analysis).

Of the remaining 4,253 variants, 260 were categorized as “pathogenic” variants, 113 were categorized as “likely pathogenic” variants, 167 variants were categorized as “benign” variants, 769 were categorized as “likely benign” variants, 1,386 were categorized as having “unknown significance” in the deafness variation database, and 1,558 were novel variants that were not identified in the deafness variation database.

Among the 260 previously reported “pathogenic” variants, 117 were identified in three or more cases among the 3,719 hearing loss patients (Supp. Table S1). With regard to the odds ratio compared with the Japanese controls (including the HGVD, 2KJPN, and in‐house controls), most of the previously reported “pathogenic” variants showed a high odds ratio concordance with the respective inheritance pattern. For example, 12 *GJB2* (MIM# 121011) mutations (the most common cause of autosomal recessive hearing loss) were included in the 117 selected variants, with eight out of the 12 showing an odds ratio of 5.0 or higher (seven out of these eight variants did not include 1.0 in the 95% confidence interval range of the odds ratio and the *P* value was under 0.05) when comparing the families with apparently autosomal recessive inheritance or sporadic cases with the control population (Supp. Table S1). These results clearly indicated the inheritance mode‐specific allele accumulation of true “pathogenic” variants through the database. As a notable result, the *GJB2*: NM_004004.5:c.368C>A:p.T123N variant revealed a low odds ratio that did not differ among the families with apparently autosomal dominant and those with apparently autosomal recessive inheritance. This variant was re‐categorized as a rare polymorphism in our previous report [Tsukada et al., 2010]. From these results, the accumulation of a large number of NGS results will provide a useful tool for the classification of variant pathogenicity. However, attention should be paid to the odds ratios calculated for single‐digit numbers of patients.

### Case Study 1

**Figure 2:**
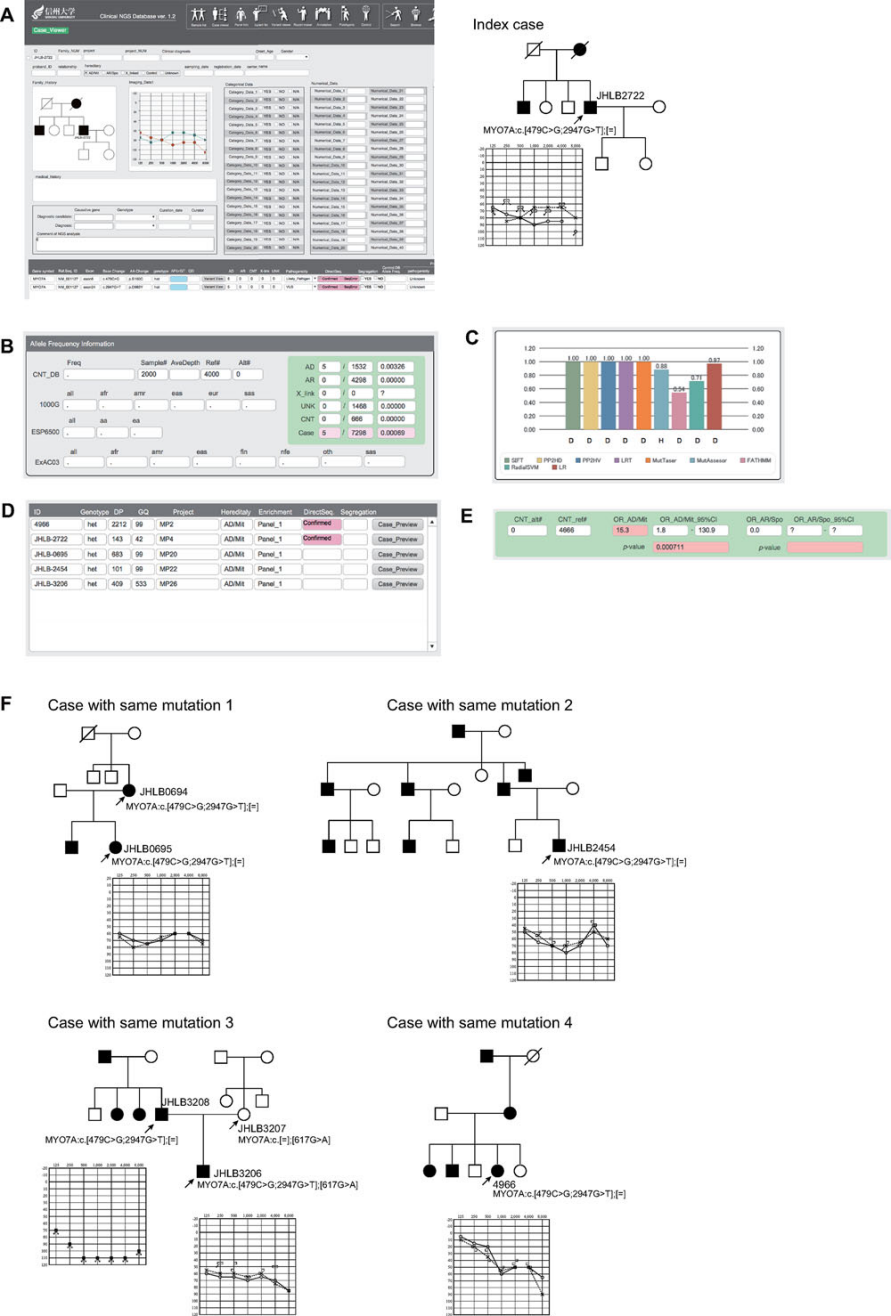
Case study of two *MYO7A* variants located in the *cis* allele. **A**: Two *MYO7A* variants were identified from the index family. **B**: Both of these mutations were novel variants and were not identified in the 1000 Genomes, ESP6500, ExAC03, dbSNP144, HGVD, or 2KJPN Project results. **C**: All in silico prediction programs predict the *MYO7A*:NM_000260:c.479C>G:p.S160C variant to be “Damaging.” **D**: By using the “Variant viewer” of the database, we could easily identify the cases carrying the same variants. **E**: The odds ratio of the variants among autosomal dominant cases was 14.1‐fold higher than the control. **F**: From the family segregation analysis of another family, these variants were found to be located in the *cis* allele and were considered to be the genetic cause of these cases autosomal dominant inherited hearing loss. See manuscript for details.

Patient 1 was a 62‐year‐old man diagnosed with sensorineural progressive hearing loss identified at school age (case number: JHLB2722) (Fig. 2 and Supp. Table S2). In his pedigree, his elder brother and his mother also suffered from hearing loss and his hearing loss was considered to be a case of autosomal dominant or mitochondrial inherited hearing loss. The target re‐sequencing analysis of this patient identified 333 variants in the 68 known deafness causing genes. Fifteen of the 333 identified variants had an MAF under 1% in 1000 Genomes, ESP6500, and ExAC databases. Nine of these 15 variants were located in the intronic or UTRs and six variants were located in the exonic region. Of these six variants, one was synonymous, leaving five remaining variants. Three of these five variants had an MAF of over 1% in the in‐house control so that, finally, only two variants remained. This variant filtering was performed using the auto‐filtering function of the Clinical NGS DB. Both of the two remaining variants were *MYO7A* (MIM# 276903) gene variants (Fig. 2A): One was *MYO7A*:NM_000260.3:c.479C>G:p.S160C and the other was *MYO7A*:NM_000260.3: c.2947G>T:p.D983Y. Both of these two mutations were novel variants and were not identified in 1000 Genomes, ESP6500, ExAC03, dbSNP144, HGVD, or 2KJPN Project results (Fig. 2B; evidence level of ACMG guidelines is indicated by the abbreviated code: PM2). Further, all in silico prediction programs predict the *MYO7A*:NM_000260.3:c.479C>G:p.S160C variant to be “Damaging” (Fig. 2C, PP3); however, some programs predict *MYO7A*:NM_000260.3: c.2947G>T:p.D983Y to be benign. It was, therefore, difficult to draw any conclusion regarding the pathogenicity of these variants from the public database and NGS results alone. We readily identified patients carrying the same variants from among our Clinical NGS DB software collection of over 4,052 samples of NGS results. Among the 4,052 cases, five patients (including this index case) carried the same variants, so we compared the NGS results and phenotypes of these cases (Fig. 2D and F). As a result, the hearing loss in all of the other cases was found to be autosomal dominant or mitochondrial inheritance and all of the patients carried both the *MYO7A* mutations. Among these cases, some had family samples, allowing us to perform segregation analysis, and we concluded that these mutations were located in the same allele (*cis* allele) and all showed a concordance segregation pattern with family history (PM7 upgraded from PP1). All of these five cases have flat or mid‐frequency dominant hearing loss and the onset age of these patients was about 10 years. The relative risk of these variants among families with apparently autosomal dominant inheritance was 14.1‐fold higher than the control (the 95% confidence interval of the odds ratio ranged from 1.78 to 130.68, and the *P* value was 0.0007), which supports the pathogenicity of these variants for autosomal dominant inherited hearing loss (Fig. 2E, PS4). This high odds ratio was only observed in families with apparently dominant inheritance and not in those with recessive inheritance, which also supports the notion that this variant is a pathogenic variant for autosomal dominant inheritance. Based on the above results, we regard these variants as “Likely pathogenic variants” for autosomal dominant inherited hearing loss according to the ACMG guidelines (PS4 + PM7 + PP3):

1. The odds ratio of these variants was 14.1‐fold higher than the control (PS4).
2. Co‐segregates with deafness in five families (PM7).
3. All in silico prediction programs support a deleterious effect of the variant (PP3).

The Clinical NGS DB was found to be a useful tool for the systematic comparison of cases carrying the same variant and was also useful for pathogenicity classification by collecting family segregation data. It is noteworthy that case number JHLB3206 carried the same variants, with the *MYO7A*:NM_000260.3:exon7:c.617G>A:p.R206H variant previously reported as a pathogenic variant [Su et al., 2009]. However, this variant (*MYO7A*:NM_000260.3:exon7:c.617G>A:p.R206H) was also identified in his normal hearing mother, suggesting it to be a rare “benign” variant, and *MYO7A*: NM_000260.3:c.[479C>G; c.2947G>T]:p.[S160C; D983Y] is the real cause of hearing loss in this patient.

### Case Study 2

**Figure 3:**
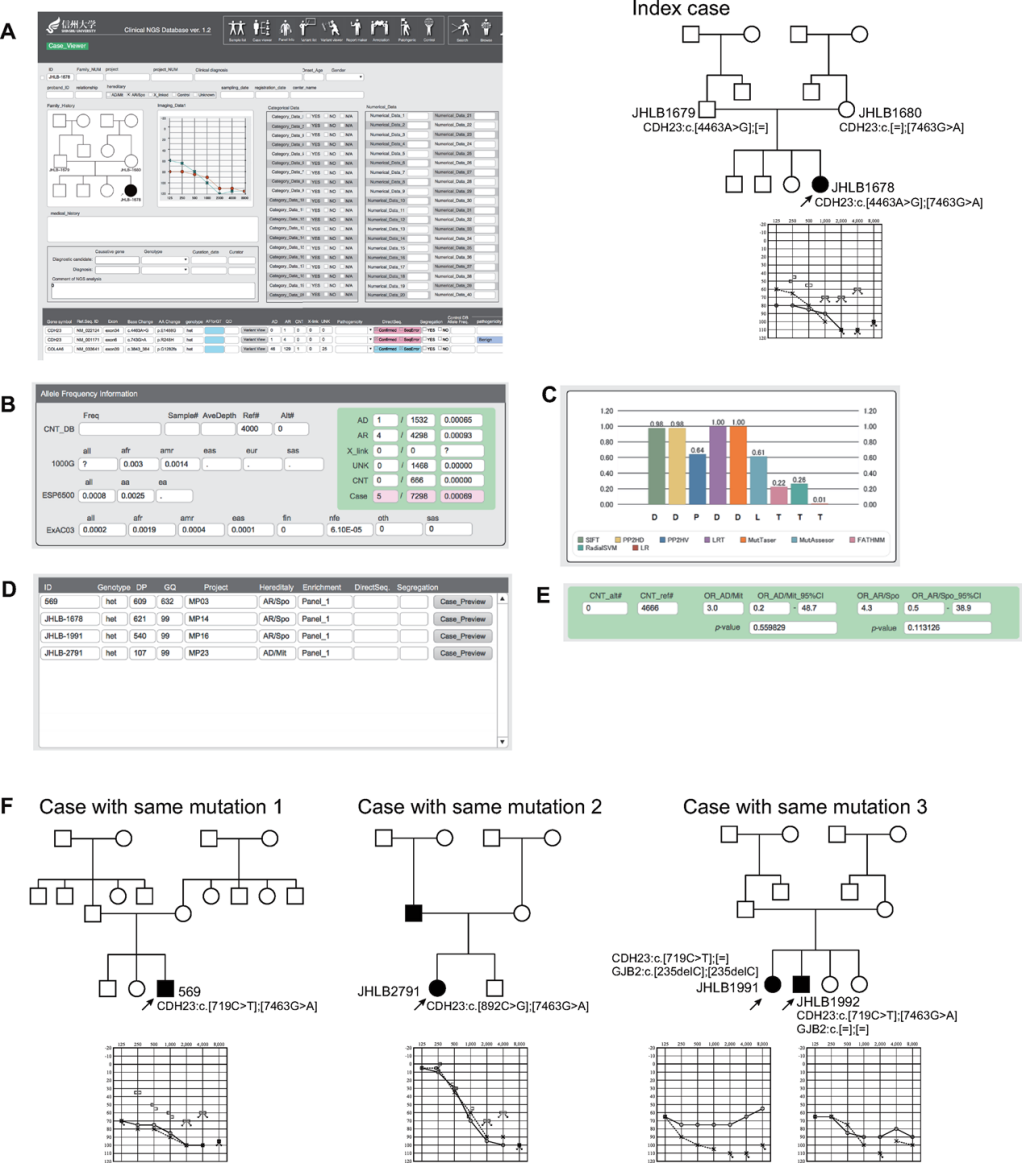
Case study of two *CDH23* variants that were identified in four cases. **A**: Two *CDH23* variants were identified from the index family. **B**: c.7463G>A was a novel variants and identified at low frequencies among the 1000 Genomes, ESP6500, ExAC03, dbSNP144, HGVD, or 2KJPN Project results. **C**: Four out of five in silico prediction programs predict this variant to be “Damaging,” but some predict it to be “Tolerant.” **D**: By using the “Variant viewer” of the database, we could easily identify the cases carrying the same variants. **E**: The odds ratio of the variants among autosomal recessive cases was 4.3‐fold higher than the control, but the difference was not significant. **F**: From the family segregation analysis of another three families, all of these cases were found to have a compound heterozygous *CDH23* variant, supporting the pathogenicity of this variant. See manuscript for details.

Patient 2 was a 12‐year‐old girl diagnosed with congenital sensorineural hearing loss identified by newborn hearing screening (case number: JHLB1678) (Fig. 3 and Supp. Table 2). In her pedigree, only she suffered hearing loss and she was considered to be a sporadic case. The target re‐sequencing analysis of this patient identified 392 variants in the 68 known deafness causing genes. Twenty‐four of the 392 identified variants had an MAF under 1% in the 1000 Genomes, ESP6500, and ExAC databases. Fourteen of these 24 variants were located in the intronic or UTRs and 10 variants were located in the exonic region. Two of these 10 variants were synonymous, so that only eight variants remained. Three of these eight variants had an MAF over 1% in the in‐house control so that, finally, five variants remained, with three of them presumed to be benign variants or polymorphisms based on other case results (Fig. 3A). Both of the two resultant variants were *CDH23* (MIM# 605516) gene variants. One variant was *CDH23*:NM_022124.5: c.4463A>G:p.E1488G and the other was *CDH23*:NM_022124.5:c.7463G>A:p.R2488H. From these results, we considered a compound heterozygous mutation of the *CDH23* gene to be the genetic cause of her hearing loss.

Among the two mutations, *CDH23*:NM_022124.5: c.4463A>G:p.E1488G was a novel variant that was not identified in the 1000 Genomes, ESP6500, ExAC03, dbSNP144, HGVD, or 2KJPN Project results. Further, all in silico prediction programs predict this variant to be “Damaging.” On the other hand, *CDH23*:NM_022124.5:c.7463G>A:p.R2488H was identified at low frequency in the 1000 Genomes, ESP6500, ExAC03, and 2KJPN Project results (Fig. 3B, PM2), and four of five in silico prediction programs predict this variant to be “Damaging” (Fig. 3C). However, a single submitter classified this variant as a “likely benign variant” in the ClinVar database. Thus, it was difficult to draw any conclusion about the pathogenicity of this variant from the public database and one sample result alone. Again, we readily identified the patients carrying the same variant from among our Clinical NGS DB software collection of over 4,052 samples of NGS results. Among the 4,052 cases, four patients (including this index case) carried the same variant, so we compared the NGS results and phenotypes of these cases (Fig. 3D and F). The relative risk of these variants among autosomal recessive cases was 4.3‐fold higher than the control (the 95% confidence interval ranged from 0.5 to 38.9, and the *P* value was 0.113, so we did not regard this information as evidence supporting the variant pathogenicity; Fig. 3E). As a result, three other cases were found to carry compound heterozygous *CDH23* mutations. Two of them carried the *CDH23*:NM_022124.5:c.7463G>A:p.R2488H variant together with the *CDH23*:NM_022124.5:c.719C>T:p.P240L variant, which was previously reported to be a pathogenic variant [Wagatsuma et al., 2007] (PM3), and the other case carried *CDH23*:NM_022124.5:c.7463G>A:p.R2488H with *CDH23*:NM_022124.5:c.892C>G:p.L298V. All of these four cases have residual hearing in low frequencies, which is characteristic of *CDH23*‐associated hearing loss. Based on the above results, we regard these variants as “likely pathogenic variants” for autosomal recessive inherited hearing according to the ACMG guidelines (PM2 + PM3 + PM7):

1. The variants were not identified in controls (PM2).
2. The variant was identified in *trans* with a pathogenic variant (PM3).
3. Co‐segregates with deafness in four families (PM7).

The Clinical NGS DB was a useful tool for browsing all data from many public databases and was also useful for the systematic comparison of each case carrying the same variant.

Interestingly, the autosomal recessive siblings (case number JHLB1991 and JHLB1992) and the proband (JHLB1992) of this family carried this variant together with the *CDH23*:NM_022124.5:exon8:c.719C>T:p.P240L variant, which was previously reported to be a pathogenic variant [Wagatsuma et al., 2007], thus we considered *CDH23* compound heterozygous mutations to be a cause of his hearing loss. Whereas his elder sister (JHLB1991) carried only heterozygous *CDH23* variants, the patient also carried a homozygous *GJB2*:NM_004004.5:exon2: c.235delC:p.L79fs mutation. So we concluded that this pedigree has two different genetic causes of hearing loss despite the fact the two cases were siblings.

## Conclusions

The interpretation of the pathogenicity of a large number of variants identified by NGS analysis is a new and important challenge in this field. Indeed, a not negligible portion of variants classified as “pathogenic variants” in public or commercial disease‐specific databases are not truly pathogenic variants [Bell et al., 2011; Shearer et al., 2014; Wang and Shen, 2014], and re‐classification of these variants is desired. However, such re‐classification requires a good deal of time and effort. Based on this database software, we propose that the phenotype similarity and odds ratio for the comparison of inheritance mode‐specific and control cases (families with apparently autosomal dominant inheritance vs. control and families with apparently autosomal recessive inheritance vs. control) can provide powerful resources for variant pathogenicity classification. This database software also represents a powerful base for the unified management of genome analysis information and patient clinical information for efficient clinical diagnosis.

## Software Availability

The Clinical NGS DB is freely available to academia at http://www.shinshu-jibi.jp/clinicalngsdb.html

*Disclosure statement*: The authors declare no conflict of interest.

## Supporting information

Disclaimer: Supplementary materials have been peer‐reviewed but not copyedited.

Supp. Figure S1 Screen shots of the case viewer.
Supp. Figure S2 Screen shots of the variant viewer.
Supp. Table S1. Previously reported pathogenic variants identified in 3,719 Japanese hearing loss patients.
Supp. Table S2. Minor allele frequency and *in silico* prediction results of the variants described in the manuscript.
