# Supplementary material for: The Clinical Next‐Generation Sequencing Database: A Tool for the Unified Management of Clinical Information and Genetic Variants to Accelerate Variant Pathogenicity Classification
Source: Hum Mutat. 2017 Jan 11;38(3):252–9. doi: 10.1002/humu.23160 (PMC5324660; doi:10.1002/humu.23160)
Supplement: Supplementary file 1 — Supp. Figure S1 Screen shots of the case viewer. Supp. Figure S2 Screen shots of the variant viewer. Supp. Table S1. Previously reported pathogenic variants identified in 3,719 Japanese hearing loss patients. Supp. Table S2. Minor allele frequency and in silico prediction results of the variants described in the manuscript. [file HUMU-38-252-s001.pdf]

Supp. Figure S1 Screen shots of the case viewer.

The case viewer is an interface for the unified management of detailed patient clinical information and variant information obtained from next-generation sequencing analysis. The variant list is automatically filtered from a large number of identified variants to the selected candidate variant.

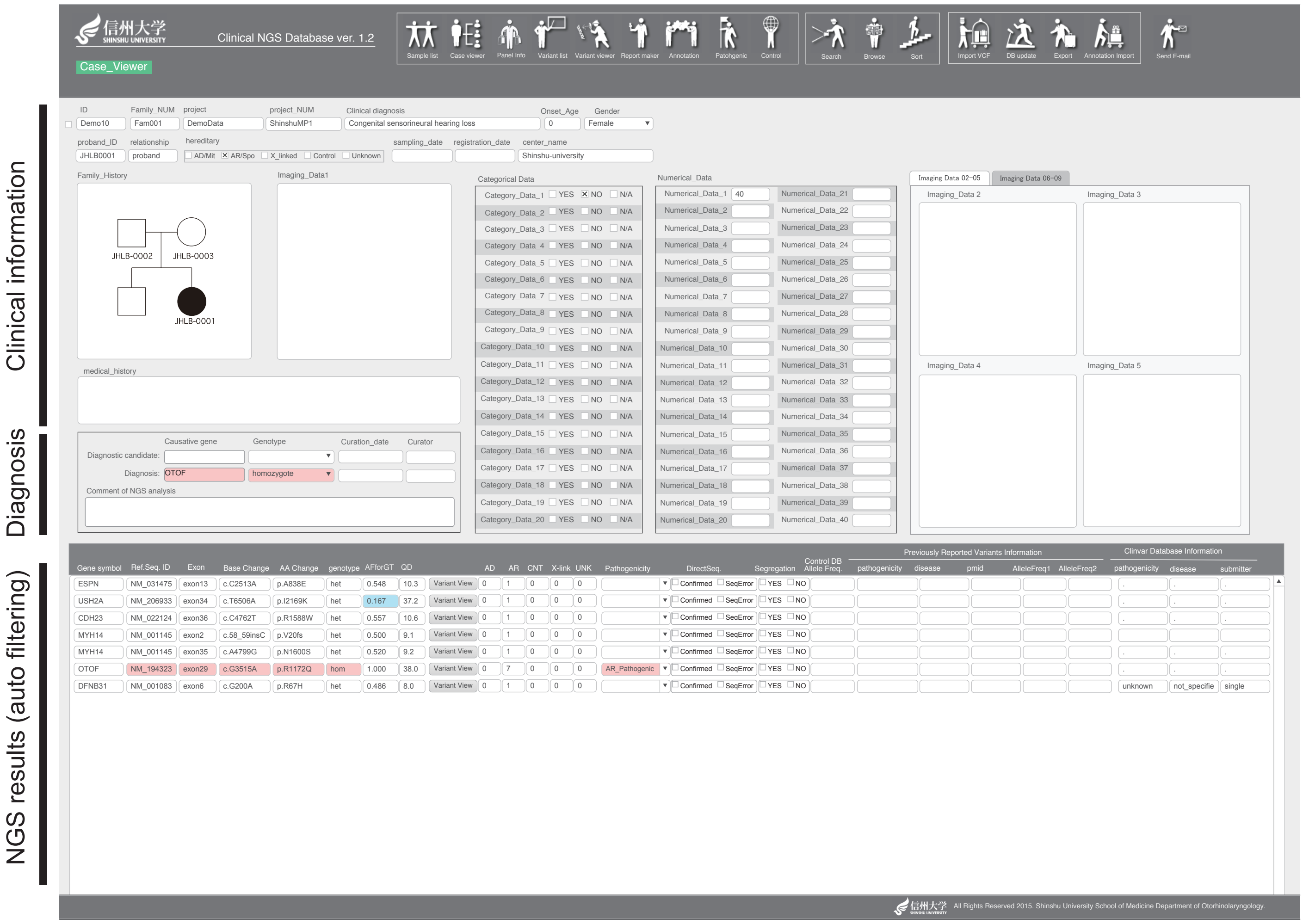

Supp. Figure S2 Screen shots of the variant viewer.

The variant viewer is an interface for accessing all knowledge regarding the variant, including the phenotypes of the variant carriers and inheritance mode-specific odds ratios. It is also useful for managing the list of patients carrying the same variant and assists in enabling time-efficient browsing of these patients.

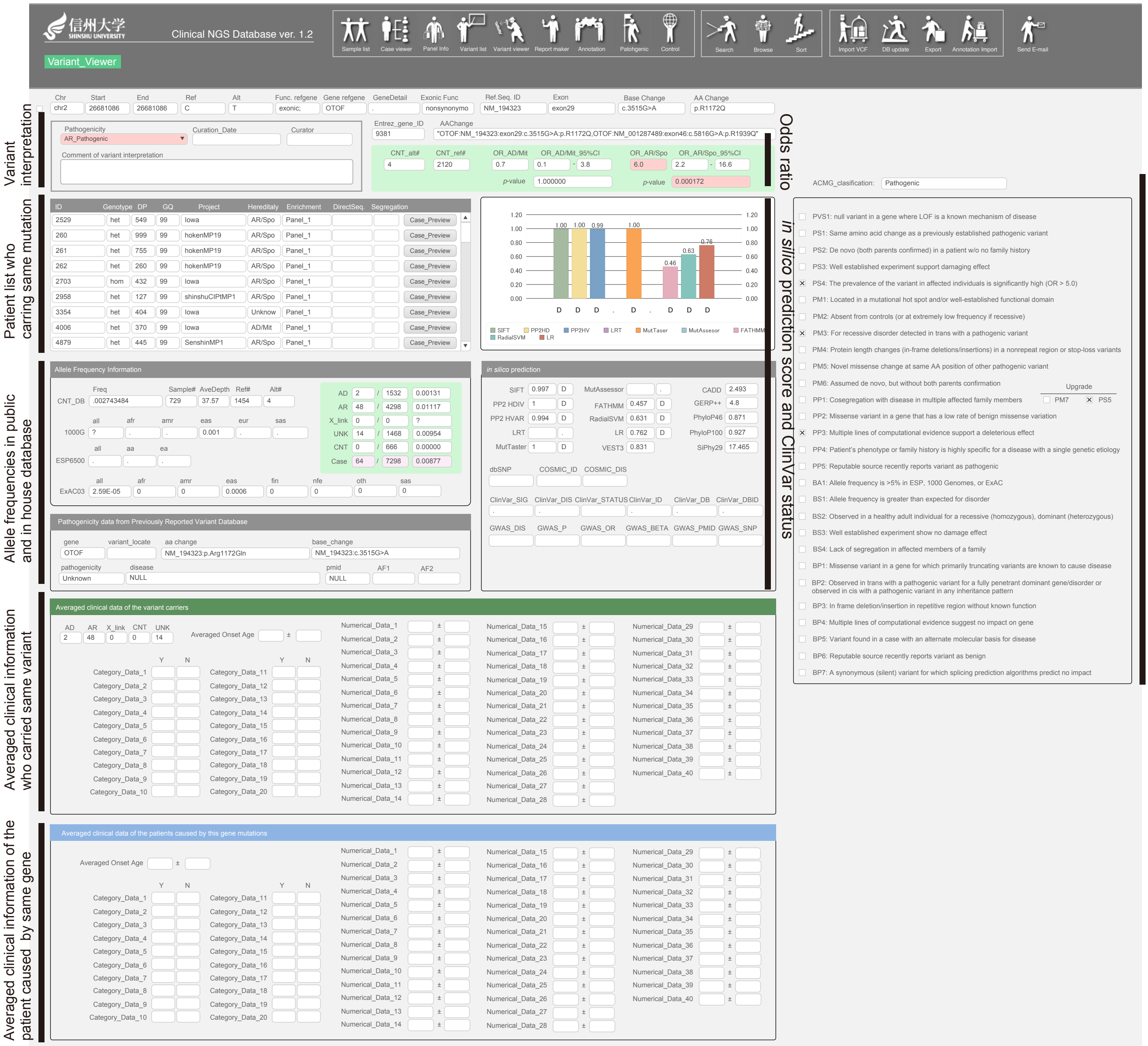

Supp. Table S1. Previously reported pathogenic variants identified in 3,719 Japanese hearing loss patients.

Supplemental Table S1. Previously reported pathogenic variants identified in 3,719 Japanese hearing loss patients.

Chr	Start*	End*	Ref	Alt	Refgene	RefSeq	Base change	AA Change	SIFT	PP2	LRT	Mut Taster	Mut Assessor	ExAC_all	ExAC_eas	Pathogenicity	AD alt	AR alt	AD ref	AR ref	JPN_CNT alt	JPN_CNT ref	AD vs. CNT OR	AR vs. CNT OR	AD vs. CNT OR_95% CI	AR vs. CNT OR_95% CI	AD vs. CNT *p* value	AR vs. CNT *p* value
17	79478023	79478023	A	G	*ACTG1*	NM_001614.3	c.914T>C	p.M305T	.	P	D	D	H	.	.	AD_Pathogenic	2	1	1662	4739	0	4690	> 5.6	> 1.0	0.5 - 62.3	0.1 - 15.8	0.116	0.996
17	79478042	79478042	G	C	*ACTG1*	NM_001614.3	c.895C>G	p.L299V	.	P	D	D	M	.	.	AD_Pathogenic	3	1	1661	4739	0	4690	> 8.5	> 1.0	0.9 - 81.5	0.1 - 15.8	0.024	0.996
17	79478939	79478939	T	A	*ACTG1*	NM_001614.3	c.353A>T	p.K118M	.	B	D	A	H	.	.	AD_Pathogenic	3	0	1661	4740	0	4690	> 8.5	> 1.0	0.9 - 81.5	NA	0.024	NA
10	73330641	73330641	C	T	*CDH23*	NM_022124.5	c.719C>T	p.P240L	D	D	D	A	L	0.00009	0.0013	AR_Pathogenic	13	91	1651	4649	24	6938	2.3	5.7	1.2 - 4.5	3.6 - 8.9	0.025	0.000
10	73464800	73464800	G	A	*CDH23*	NM_022124.5	c.2866G>A	p.E956K	.	D	D	D	M	0.00003	0	AR_Pathogenic	2	15	1662	4725	2	4728	2.8	7.5	0.4 - 20.2	1.7 - 32.8	0.601	0.004
10	73492049	73492049	G	A	*CDH23*	NM_022124.5	c.4021G>A	p.D1341N	.	D	D	A	H	0.00001	0	AR_Pathogenic	0	4	1664	4736	0	4690	0.0	> 4.0	NA	0.4 - 35.5	NA	0.136
10	73498294	73498294	C	T	*CDH23*	NM_022124.5	c.4249C>T	p.R1417W	.	D	N	D	M	0.00009	0.0013	AR_Pathogenic	1	9	1663	4731	11	6309	0.3	1.1	0.0 - 2.7	0.5 - 2.6	0.476	0.974
10	73498391	73498391	G	A	*CDH23*	NM_022124.5	c.4346G>A	p.G1449D	.	D	D	D	M	0.00003	0.0004	AR_Pathogenic	1	11	1663	4729	12	6936	0.3	1.3	0.0 - 2.7	0.6 - 3.0	0.477	0.618
10	73538025	73538025	A	C	*CDH23*	NM_022124.5	c.5147A>C	p.Q1716P	.	D	D	D	M	.	.	AR_Pathogenic	0	4	1664	4736	0	4690	0.0	> 4.0	NA	0.4 - 35.5	NA	0.136
10	73550924	73550924	C	T	*CDH23*	NM_022124.5	c.6085C>T	p.R2029W	.	D	D	D	H	0.00003	0.0002	AR_Pathogenic	9	31	1655	4709	13	6869	2.9	3.5	1.2 - 6.7	1.8 - 6.7	0.023	0.000
10	73553004	73553004	C	T	*CDH23*	NM_022124.5	c.6319C>T	p.R2107X	.	.	D	A	.	.	.	AR_Pathogenic	0	4	1664	4736	0	4690	0.0	> 4.0	NA	0.4 - 35.5	NA	0.136
10	73558125	73558125	A	-	*CDH23*	NM_022124.5	c.6844delA	p.N2282fs	.	.	.	.	.	.	.	AR_Pathogenic	1	3	1663	4737	0	666	> 0.4	> 0.4	0.0 - 6.4	0.0 - 4.1	0.635	0.819
10	73558958	73558958	G	A	*CDH23*	NM_022124.5	c.7145G>A	p.R2382Q	D	D	D	D	L	0.00002	0.0003	AR_Pathogenic	1	5	1663	4735	4	6162	0.9	1.6	0.1 - 8.3	0.4 - 6.1	0.632	0.692
10	73559336	73559336	G	A	*CDH23*	NM_022124.5	c.7312G>A	p.E2438K	T	B	D	D	N	.	.	AR_Pathogenic	4	3	1660	4737	0	4690	> 11.3	> 3.0	1.3 -101.2	0.3 - 28.6	0.005	0.252
16	21270106	21270106	T	G	*CRYM*	NM_001888.4	c.941A>C	p.K314T	D	P	D	A	N	.	.	AD_Pathogenic	0	2	1664	4738	1	4763	0.0	2.0	NA	0.2 - 22.2	0.582	0.997
14	76964643	76964643	C	T	*ESRRB*	NM_004452.3	c.1144C>T	p.R382C	T	D	D	D	L	0.00070	0.0097	AR_Pathogenic	13	25	1651	4715	44	6852	1.2	0.8	0.7 - 2.3	0.5 - 1.4	0.634	0.522
8	72211384	72211384	T	C	*EYA1*	NM_172060.3	c.625A>G	p.S209G	T	B	D	D	M	0.00002	0.0002	AD_Pathogenic	1	3	371	827	51	6439	0.3	0.5	0.0 - 2.5	0.1 - 1.5	0.417	0.259
8	72211437	72211437	C	A	*EYA1*	NM_172060.3	c.572G>T	p.G191V	D	P	D	D	N	0.00010	0.0018	AD_Pathogenic	2	3	370	827	62	6486	0.6	0.4	0.1 - 2.3	0.1 - 1.2	0.601	0.133
8	72233984	72233984	C	T	*EYA1*	NM_172060.3	c.304G>A	p.G102S	T	D	D	D	N	0.00007	0.0008	AD_Pathogenic	3	2	369	828	21	5775	2.2	0.7	0.7 - 7.5	0.2 - 2.8	0.366	0.810
13	20763138	20763138	T	C	*GJB2*	NM_004004.5	c.583A>G	p.M195V	D	D	D	D	M	0.00003	0.0002	AD_Pathogenic	1	6	1663	4734	1	5547	3.3	7.0	0.2 - 53.5	0.8 - 58.4	0.948	0.084
13	20763209	20763209	-	CGTT	*GJB2*	NM_004004.5	c.511_512insAACG	p.A171fs	.	.	.	.	.	0.00002	0.0002	AR_Pathogenic	0	14	1664	4726	0	666	0.0	> 2.0	NA	0.3 - 15.0	NA	0.319
13	20763294	20763294	G	A	*GJB2*	NM_004004.5	c.427C>T	p.R143W	D	D	D	A	M	0.00020	0.0002	AR_Pathogenic	9	72	1655	4668	7	5599	4.3	12.3	1.6 - 11.7	5.7 - 26.8	0.004	0.000
13	20763313	20763313	G	T	*GJB2*	NM_004004.5	c.408C>A	p.Y136X	.	.	D	D	.	.	.	AR_Pathogenic	11	106	1653	4634	16	7026	2.9	10.0	1.4 - 6.3	5.9 - 17.0	0.009	0.000
13	20763353	20763353	G	T	*GJB2*	NM_004004.5	c.368C>A	p.T123N	T	B	N	N	N	0.00050	0.0073	AR_Pathogenic	8	19	1656	4721	46	6986	0.7	0.6	0.3 - 1.6	0.4 - 1.0	0.525	0.091
13	20763421	20763422	AT	-	*GJB2*	NM_004004.5	c.299_300del	p.H100fs	.	.	.	.	.	0.00004	0.0006	AR_Pathogenic	2	47	1662	4693	2	1522	0.9	7.6	0.1 - 6.5	1.8 - 31.4	0.680	0.002
13	20763464	20763464	G	C	*GJB2*	NM_004004.5	c.257C>G	p.T86R	D	D	D	D	M	.	.	AR_Pathogenic	6	21	1658	4719	4	6289	5.7	7.0	1.6 - 20.2	2.4- 20.4	0.008	0.000
13	20763486	20763486	G	-	*GJB2*	NM_004004.5	c.235delC	p.L79fs	.	.	.	.	.	0.00040	0.005	AR_Pathogenic	26	452	1638	4288	27	2123	1.2	8.3	0.7 - 2.1	5.6 - 12.3	0.507	0.000
13	20763530	20763545	CACACGTT CTTGCAGC	-	*GJB2*	NM_004004.5	c.176_191del	p.G59fs	.	.	.	.	.	0.00002	0.0002	AR_Pathogenic	5	58	1659	4682	0	666	> 2.0	> 8.3	0.2 - 17.2	1.1 - 59.7	0.357	0.008
13	20763587	20763587	C	T	*GJB2*	NM_004004.5	c.134G>A	p.G45E	D	D	D	A	M	.	.	AR_Pathogenic	11	106	1653	4634	16	7018	2.9	10.0	1.4 - 6.3	5.9 - 17.0	0.009	0.000
13	20763612	20763612	C	T	*GJB2*	NM_004004.5	c.109G>A	p.V37I	T	D	D	A	M	0.00660	0.0724	AR_Pathogenic	27	101	1637	4639	67	6891	1.7	2.2	1.1 -2.7	1.6 - 3.1	0.028	0.000
13	20763698	20763698	G	A	*GJB2*	NM_004004.5	c.23C>T	p.T8M	D	P	D	N	L	0.00007	0	AR_Pathogenic	1	1	1663	4739	10	6166	0.4	0.1	0.0 - 2.9	0.0 - 1.0	0.538	0.046
1	35250822	35250822	G	T	*GJB3*	NM_024009.2	c.459G>T	p.W153C	D	D	D	D	M	.	.	AD_Pathogenic	3	8	1661	4732	2	5496	5.0	4.6	0.8 - 29.7	1.0 - 21.9	0.156	0.069
13	20796930	20796930	-	T	*GJB6*	NM_006783.4	c.689dupA	p.N230fs	.	.	.	.	.	0.00030	0	AR_Pathogenic	4	5	1660	4735	0	666	> 1.6	> 0.7	0.2 -14.4	0.1 - 6.0	0.476	0.875
13	20797319	20797319	C	T	*GJB6*	NM_006783.4	c.301G>A	p.E101K	T	B	D	D	N	0.00010	0.0001	AR_Pathogenic	0	2	1664	4738	1	4761	0.0	2.0	NA	0.2 - 22.2	0.582	0.997
1	41249975	41249975	C	-	*KCNQ4*	NM_004700.3	c.210delC	p.G70fs	.	.	.	.	.	.	.	AD_Pathogenic	25	0	1639	4740	0	666	> 10.2	0.0	1.4 - 75.1	NA	0.003	NA
1	41284190	41284190	C	G	*KCNQ4*	NM_004700.3	c.546C>G	p.F182L	T	B	N	A	N	0.00030	0.0045	AD_Pathogenic	3	16	1661	4724	32	5928	0.3	0.6	0.1 - 1.1	0.3 - 1.1	0.090	0.165
1	41304121	41304121	G	A	*KCNQ4*	NM_004700.3	c.2014G>A	p.V672M	D	D	N	D	N	0.00006	0.0008	AD_Pathogenic	0	3	1664	4737	3	4707	0.0	1.0	NA	0.2 - 4.9	0.710	0.689
18	44109190	44109190	G	A	*LOXHD1*	NM_144612.6	c.4480C>T	p.R1494X	.	.	D	A	.	0.00060	0	AR_Pathogenic	0	3	1664	4737	1	4745	0.0	3.0	NA	0.3 - 28.9	0.583	0.616
11	71817106	71817106	C	T	*LRTOMT*	NM_001145310.3	c.88C>T	p.R30X	.	.	.	A	.	.	.	AR_Pathogenic	0	4	1664	4736	0	4690	0.0	> 4.0	NA	0.4 - 35.5	NA	0.136
5	68728503	68728503	G	A	*MARVELD2*	NM_001244734.1	c.1295+1G>A		.	.	.	D	.	0.00002	0	AR_Pathogenic	0	3	1664	4737	0	4690	0.0	> 3.0	NA	0.3 - 28.6	NA	0.252
5	68736316	68736316	A	-	*MARVELD2*	NM_001244734.1	c.1507delA	p.K503fs	.	.	.	.	.	0.00003	0.0004	AR_Pathogenic	0	4	1664	4736	8	658	0.0	0.1	NA	0.0 - 0.2	0.000	0.000
5	68737445	68737445	T	A	*MARVELD2*	NM_001244734.1	c.1605T>A	p.D535E	T	D	D	D	M	0.00001	.	AR_Pathogenic	1	4	1663	4736	6	5612	0.6	0.8	0.1 - 4.7	0.2 - 2.8	0.929	0.961
19	50792843	50792843	G	A	*MYH14*	NM_001077186.1	c.4804G>A	p.E1602K	D	D	.	D	M	0.00010	0.0019	AD_Pathogenic	3	22	1661	4718	19	6363	0.6	1.6	0.2 - 2.0	0.8 - 2.9	0.580	0.203
22	36698709	36698709	G	A	*MYH9*	NM_002473.5	c.2404C>T	p.R802W	D	D	D	D	M	0.00007	0.0005	AD_Pathogenic	1	6	1663	4734	2	5264	1.6	3.3	0.1 - 17.5	0.7 - 16.5	0.766	0.226
22	36702021	36702021	C	T	*MYH9*	NM_002473.5	c.2114G>A	p.R705H	D	D	D	A	H	.	.	AD_Pathogenic	4	0	1660	4740	0	4690	> 11.3	0.0	1.3 - 101.2	NA	0.005	NA
17	18022785	18022785	A	G	*MYO15A*	NM_016239.3	c.671A>G	p.Y224C	D	D	.	N	L	0.00003	0.0004	AR_Pathogenic	6	16	1658	4724	31	6885	0.8	0.8	0.3 - 1.9	0.4 - 1.4	0.778	0.437
17	18042234	18042234	G	T	*MYO15A*	NM_016239.3	c.5117G>T	p.G1706V	D	D	.	D	M	.	.	AR_Pathogenic	0	2	1664	4738	1	4707	0.0	2.0	NA	0.2 - 21.9	0.587	0.995
17	18046936	18046936	G	A	*MYO15A*	NM_016239.3	c.5964+3G>A		.	.	.	.	.	0.00004	.	AR_Pathogenic	0	2	1664	4738	0	4690	0.0	> 2.0	NA	0.2 - 21.8	NA	0.484
17	18064657	18064657	T	A	*MYO15A*	NM_016239.3	c.9413T>A	p.L3138Q	D	D	.	D	H	.	.	AR_Pathogenic	2	3	1662	4737	0	4690	> 5.6	> 3.0	0.5 - 62.3	0.3 - 28.6	0.116	0.252
17	18075505	18075507	CTT	-	*MYO15A*	NM_016239.3	c.10251_10253del	p.3417_3418del	.	.	.	.	.	.	.	AR_Pathogenic	0	4	1664	4736	3	667	0.0	0.2	NA	0.0 - 0.8	0.036	0.061
17	18036540	18036540	G	T	*MYO15A*	NM_016239.3	c.4322G>T	p.G1441V	D	D	.	D	M	0.00001	0.0001	AR_Pathogenic	1	5	1663	4735	3	4637	0.9	1.6	0.1 - 8.9	0.4 - 6.8	0.614	0.746
10	26315356	26315356	A	C	*MYO3A*	NM_017433.4	c.848A>C	p.Q283P	T	P	D	D	M	0.00007	0.0009	AR_Pathogenic	4	14	1660	4726	13	4879	0.9	1.1	0.3 - 2.8	0.5 - 2.4	0.918	0.935
6	76623836	76623836	C	T	*MYO6*	NM_004999.3	c.3496C>T	p.R1166X	.	.	D	A	.	.	.	AD_Pathogenic	4	2	1660	4738	0	4690	> 11.3	> 2.0	1.3 - 101.2	0.2 - 21.8	0.005	0.484
11	76853753	76853753	A	G	*MYO7A*	NM_000260.3	c.19-2A>G		.	.	.	D	.	.	.	AR_Pathogenic	2	3	1662	4737	0	4690	> 5.6	> 3.0	0.5 - 62.3	0.3 - 28.6	0.116	0.252
11	76867967	76867967	G	A	*MYO7A*	NM_000260.3	c.652G>A	p.D218N	D	D	D	D	M	0.00003	0	AD_Pathogenic	1	1	1663	4739	0	4690	> 2.8	> 1.0	0.2 - 45.1	0.1 - 15.8	0.588	0.996
11	76883793	76883793	G	-	*MYO7A*	NM_000260.3	c.1798-1G>-		.	.	.	.	.	.	.	AR_Pathogenic	3	4	1661	4736	0	666	> 1.2	> 0.6	0.1 - 11.6	0.1 -5.0	0.648	0.991
11	76885889	76885889	C	T	*MYO7A*	NM_000260.3	c.2023C>T	p.R675C	D	D	D	D	H	0.00003	0.0002	AR_Pathogenic	5	4	1659	4736	1	5289	15.9	4.5	1.9 - 136.5	0.5 - 40.0	0.003	0.308
11	76886438	76886438	C	A	*MYO7A*	NM_000260.3	c.2115C>A	p.C705X	.	.	D	A	.	0.00002	0.0002	AR_Pathogenic	0	4	1664	4736	2	5550	0.0	2.3	NA	0.4 - 12.8	0.948	0.546
11	76890119	76890119	G	T	*MYO7A*	NM_000260.3	c.2311G>T	p.A771S	D	P	D	D	M	.	.	AR_Pathogenic	3	9	1661	4731	17	6311	0.7	0.7	0.2 - 2.3	0.3 - 1.6	0.714	0.517
11	76912561	76912561	G	A	*MYO7A*	NM_000260.3	c.4921G>A	p.E1641K	T	D	D	D	M	0.00004	0.0002	AR_Pathogenic	0	4	1664	4736	4	5478	0.0	1.2	NA	0.3 - 4.6	0.610	0.882
11	76916643	76916643	C	T	*MYO7A*	NM_000260.3	c.5617C>T	p.R1873W	D	D	D	D	M	0.00001	0	AR_Pathogenic	0	2	1664	4738	0	4690	0.0	> 2.0	NA	0.2 - 21.8	NA	0.484
2	26683865	26683865	C	T	*OTOF*	NM_194248.2	c.5567G>A	p.R1856Q	D	D	D	D	M	0.00001	0.0001	AR_Pathogenic	0	4	1664	4736	0	4690	0.0	> 4.0	NA	0.4 - 35.5	NA	0.136
2	26705431	26705431	A	T	*OTOF*	NM_194248.2	c.1422T>A	p.Y474X	.	.	D	A	.	.	.	AR_Pathogenic	0	8	1664	4732	0	4690	0.0	> 7.9	NA	1.0 - 63.4	NA	0.014
2	26707353	26707353	A	T	*OTOF*	NM_194248.2	c.1194T>A	p.D398E	T	B	D	D	L	0.00020	0.0029	AR_Pathogenic	4	16	1660	4724	23	6875	0.7	1.0	0.2 - 2.1	0.5 - 1.9	0.716	0.900
10	55617017	55617017	C	T	*PCDH15*	NM_001142763.1	c.3739G>A	p.V1247M	D	D	.	D	M	0.00040	0.0054	AR_Pathogenic	7	9	1657	4731	8	6164	3.3	1.5	1.2 - 9.0	0.6 - 3.8	0.036	0.585
10	56138562	56138562	C	T	*PCDH15*	NM_001142763.1	c.313G>A	p.G105R	D	D	.	D	L	0.00010	0	AR_Pathogenic	4	6	1660	4734	9	5609	1.5	0.8	0.5 - 4.9	0.3 - 2.2	0.726	0.850
7	107303801	107303801	C	G	*SLC26A4*	NM_000441.1	c.225C>G	p.L75L	.	.	.	.	.	0.00030	0.0012	AR_Pathogenic	1	5	1663	4735	6	5690	0.6	1.0	0.1 - 4.7	0.3 - 3.3	0.940	0.764
7	107312645	107312645	C	T	*SLC26A4*	NM_000441.1	c.367C>T	p.P123S	D	D	D	D	M	.	.	AR_Pathogenic	0	3	1664	4737	1	5547	0.0	3.5	NA	0.4 - 33.8	0.523	0.510
7	107314632	107314632	A	G	*SLC26A4*	NM_000441.1	c.439A>G	p.M147V	D	D	D	D	M	0.00001	0.0001	AR_Pathogenic	1	4	1663	4736	2	5362	1.6	2.3	0.1 - 17.8	0.4 - 12.4	0.775	0.575
7	107314794	107314794	G	T	*SLC26A4*	NM_000441.1	c.600+1G>T		.	.	.	D	.	0.00001	0	AR_Pathogenic	0	7	1664	4733	0	4690	0.0	> 6.9	NA	0.9 - 56.4	NA	0.024
7	107315389	107315389	G	A	*SLC26A4*	NM_000441.1	c.601-1G>A		.	.	.	D	.	.	.	AR_Pathogenic	0	4	1664	4736	4	5608	0.0	1.2	NA	0.3 - 4.7	0.621	0.908
7	107315546	107315546	A	G	*SLC26A4*	NM_000441.1	c.757A>G	p.I253V	T	P	D	D	L	0.00007	0.0007	AR_Pathogenic	1	5	1663	4735	4	5432	0.8	1.4	0.1 - 7.3	0.4 - 5.3	0.729	0.837
7	107323898	107323898	A	G	*SLC26A4*	NM_000441.1	c.919-2A>G		.	.	.	D	.	0.00030	0.0043	AR_Pathogenic	0	27	1664	4713	3	5505	0.0	10.5	NA	3.2 - 34.7	0.789	0.000
7	107323983	107323983	G	A	*SLC26A4*	NM_000441.1	c.1001+1G>A		.	.	.	A	.	0.00020	0	AR_Pathogenic	1	2	1663	4738	1	4763	2.9	2.0	0.2 - 45.8	0.2 - 22.2	0.977	0.997
7	107329611	107329611	C	T	*SLC26A4*	NM_000441.1	c.1115C>T	p.A372V	D	D	D	A	M	.	.	AR_Pathogenic	0	8	1664	4732	0	4690	0.0	> 7.9	NA	1.0 - 63.4	NA	0.014
7	107330593	107330593	A	T	*SLC26A4*	NM_000441.1	c.1174A>T	p.N392Y	D	D	D	D	M	0.00001	0.0001	AR_Pathogenic	0	7	1664	4733	1	4763	0.0	7.0	NA	0.9 - 57.3	0.582	0.076
7	107330645	107330645	G	A	*SLC26A4*	NM_000441.1	c.1226G>A	p.R409H	D	D	D	D	M	0.00010	0	AR_Pathogenic	0	3	1664	4737	0	4690	0.0	> 3.0	NA	0.3 - 28.6	NA	0.252
7	107330648	107330648	C	T	*SLC26A4*	NM_000441.1	c.1229C>T	p.T410M	D	D	D	D	M	0.00020	0	AR_Pathogenic	0	26	1664	4714	3	5501	0.0	10.1	NA	3.1 - 33.4	0.788	0.000
7	107334899	107334899	G	A	*SLC26A4*	NM_000441.1	c.1315G>A	p.G439R	D	D	N	D	M	0.00002	0	AR_Pathogenic	0	4	1664	4736	2	4688	0.0	2.0	NA	0.4 - 10.8	0.970	0.693
7	107336429	107336429	G	A	*SLC26A4*	NM_000441.1	c.1489G>A	p.G497S	D	D	D	A	M	0.00002	0.0001	AR_Pathogenic	1	2	1663	4738	0	4690	> 2.8	> 2.0	0.2 - 45.1	0.2 - 21.8	0.588	0.484
7	107338521	107338521	A	C	*SLC26A4*	NM_000441.1	c.1579A>C	p.T527P	D	D	D	D	M	.	.	AR_Pathogenic	0	6	1664	4734	1	5435	0.0	6.9	NA	0.8 - 57.2	0.531	0.090
7	107340560	107340560	-	T	*SLC26A4*	NM_000441.1	c.1648dupT	p.R549fs	.	.	.	.	.	.	.	AR_Pathogenic	1	6	1663	4734	2	2122	0.6	1.3	0.1 - 7.0	0.3 - 6.7	0.832	0.985
7	107340625	107340625	G	A	*SLC26A4*	NM_000441.1	c.1707+5G>A		.	.	.	.	.	0.00003	0.0002	AR_Pathogenic	0	11	1664	4729	0	4690	0.0	> 10.9	NA	1.4 - 84.5	NA	0.003
7	107342443	107342443	G	C	*SLC26A4*	NM_000441.1	c.1975G>C	p.V659L	D	P	D	D	L	0.00001	0.0001	AR_Pathogenic	0	6	1664	4734	0	4690	0.0	> 5.9	NA	0.7 - 49.4	NA	0.042
7	107344815	107344815	T	C	*SLC26A4*	NM_000441.1	c.2074T>C	p.F692L	T	P	D	D	N	.	.	AR_Pathogenic	1	5	1663	4735	1	5435	3.3	5.7	0.2 - 52.3	0.7 - 49.1	0.958	0.163
7	107350514	107350514	-	GCTGG	*SLC26A4*	NM_000441.1	c.2105_2106insGCTGG	p.K702fs	.	.	.	.	.	.	.	AR_Pathogenic	0	4	1664	4736	0	666	0.0	> 0.6	NA	0.1 - 5.0	NA	0.991
7	107350571	107350571	C	T	*SLC26A4*	NM_000441.1	c.2162C>T	p.T721M	D	D	D	A	M	0.00006	0	AR_Pathogenic	1	8	1663	4732	0	4690	> 2.8	> 7.9	0.2 - 45.1	1.0 - 63.4	0.588	0.014
7	107350577	107350577	A	G	*SLC26A4*	NM_000441.1	c.2168A>G	p.H723R	D	D	D	A	M	0.00010	0.0017	AR_Pathogenic	9	125	1655	4615	16	6952	2.4	11.8	1.0 - 5.4	7.0 - 19.8	0.062	0.000
7	107323796	107323796	-	G	*SLC26A4*	NM_000441.1	c.916dupG	p.I305fs	.	.	.	.	.	0.00002	0.0002	AR_Pathogenic	0	3	1664	4737	2	2122	0.0	0.7	NA	0.1 - 4.0	0.590	0.964
7	107323901	107323901	C	T	*SLC26A4*	NM_000441.1	c.920C>T	p.T307M	D	D	D	D	L	0.00006	0	AR_Pathogenic	0	4	1664	4736	3	5617	0.0	1.6	NA	0.4 - 7.1	0.799	0.821
15	43893673	43893673	C	T	*STRC*	NM_153700.2	c.4622G>A	p.R1541Q	T	P	N	D	.	0.00020	0.0027	AR_Pathogenic	3	4	1565	4514	7	5457	1.5	0.7	0.4 - 5.8	0.2 - 2.4	0.837	0.772
11	121023682	121023682	C	T	*TECTA*	NM_005422.2	c.4198C>T	p.H1400Y	T	D	D	D	N	0.00020	0.0027	AD_Pathogenic	0	3	1664	4737	11	5463	0.0	0.3	NA	0.1 - 1.1	0.141	0.108
11	121038844	121038844	C	T	*TECTA*	NM_005422.2	c.5668C>T	p.R1890C	D	D	D	A	L	.	.	AD_Pathogenic	2	1	1662	4739	0	4690	> 5.6	> 1.0	0.5 - 62.3	0.1 - 15.8	0.116	0.996
9	71869279	71869279	A	G	*TJP2*	NM_004817.3	c.3562A>G	p.T1188A	D	D	D	D	L	0.00009	0.0013	AD_Pathogenic	8	36	1608	4588	48	6890	0.7	1.1	0.3 - 1.5	0.7 - 1.7	0.476	0.670
21	43796685	43796685	C	T	*TMPRSS3*	NM_032404.2	c.778G>A	p.A260T	D	D	D	D	L	0.00003	0.0001	AR_Pathogenic	0	7	1664	4733	1	4633	0.0	6.9	NA	0.8 - 55.5	0.593	0.082
21	43809080	43809080	C	T	*TMPRSS3*	NM_032405.1	c.280G>A	p.G94R	D	D	D	D	M	0.00010	0.0002	AR_Pathogenic	11	30	1653	4710	50	6960	0.9	0.9	0.5 - 1.8	0.6 - 1.4	0.947	0.685
22	38106473	38106473	G	A	*TRIOBP*	NM_001039141.2	c.154G>A	p.D52N	D	D	.	N	L	0.00020	0.0016	AR_Pathogenic	4	11	1660	4729	13	6093	1.1	1.1	0.4 - 3.5	0.5 - 2.4	0.934	0.996
22	38167667	38167667	G	A	*TRIOBP*	NM_007032.5	c.1721G>A	p.R574H	D	D	.	D	M	.	.	AR_Pathogenic	2	4	1662	4736	5	4619	1.1	0.8	0.2 - 5.7	0.2 - 2.9	0.763	0.970
11	17553006	17553006	C	T	*USH1C*	NM_005709.3	c.188G>A	p.R63Q	D	D	D	D	L	0.00001	0	AR_Pathogenic	4	1	1660	4739	0	4690	> 11.3	> 1.0	1.3 - 101.2	0.1 - 15.8	0.005	0.996
1	215844430	215844430	A	G	*USH2A*	NM_206933.2	c.14017T>C	p.Y4673H	T	P	U	D	M	.	.	AR_Pathogenic	4	14	1518	4344	18	5422	0.8	1.0	0.3 -2.3	0.5 - 2.0	0.873	0.924
1	216051224	216051224	T	C	*USH2A*	NM_206933.2	c.8559-2A>G		.	.	.	D	.	0.00002	0.0003	AR_Pathogenic	4	5	1518	4353	0	4512	> 11.9	> 5.2	1.3 - 106.5	0.6 - 44.4	0.004	0.067
1	216138779	216138779	T	C	*USH2A*	NM_206933.2	c.7000A>G	p.N2334D	T	P	N	N	L	0.00009	0.0013	AR_Pathogenic	3	1	1519	4357	2	5178	5.1	0.6	0.9 - 30.6	0.1 - 6.6	0.145	0.881
1	216246607	216246607	G	A	*USH2A*	NM_206933.2	c.5608C>T	p.R1870W	D	D	N	D	M	0.00040	0.0049	AR_Pathogenic	2	7	1520	4351	22	6164	0.4	0.5	0.1 - 1.6	0.2 - 1.1	0.250	0.090
1	216348605	216348605	G	A	*USH2A*	NM_206933.2	c.4616C>T	p.T1539I	D	D	U	D	M	0.00008	0.0011	AR_Pathogenic	0	3	1522	4355	4	6174	0.0	1.1	NA	0.2 - 4.8	0.715	0.761
1	216371668	216371668	G	A	*USH2A*	NM_206933.2	c.4070C>T	p.T1357M	D	D	U	D	M	0.00020	0.0013	AR_Pathogenic	7	26	1515	4332	46	6820	0.7	0.9	0.3 - 1.5	0.5 - 1.4	0.449	0.724
1	216462716	216462716	C	T	*USH2A*	NM_206933.2	c.1877G>A	p.R626Q	D	D	N	D	M	0.00006	0.0005	AR_Pathogenic	0	3	1522	4355	0	4512	0.0	> 3.1	NA	0.3 - 29.9	NA	0.236
1	216465679	216465679	G	C	*USH2A*	NM_206933.2	c.1678C>G	p.P560A	T	P	U	D	M	0.00006	0.0007	AR_Pathogenic	0	3	1522	4355	3	4583	0.0	1.1	NA	0.2 - 5.2	0.741	0.729
1	216500979	216500979	C	T	*USH2A*	NM_206933.2	c.802G>A	p.G268R	D	D	U	D	M	0.00001	0	AR_Pathogenic	2	1	1520	4357	0	4512	> 5.9	> 1.0	0.5 - 62.3	0.1 - 16.6	0.105	0.986
4	6303573	6303573	C	T	*WFS1*	NM_006005.3	c.2051C>T	p.A684V	D	D	D	D	M	.	.	AD_Pathogenic	1	5	1663	4735	0	4690	> 2.8	> 5.0	0.2 - 45.1	0.6 - 42.4	0.588	0.075
4	6303641	6303641	G	T	*WFS1*	NM_006005.3	c.2119G>T	p.V707F	D	D	D	D	M	.	.	AD_Pathogenic	2	0	1662	4740	0	4690	> 5.6	0.0	0.5 -62.3	NA	0.116	NA
4	6303644	6303644	C	T	*WFS1*	NM_006005.3	c.2122C>T	p.R708C	D	D	D	D	M	0.00010	0.0002	AR_Pathogenic	0	4	1664	4736	6	5602	0.0	0.8	NA	0.2 - 2.8	0.396	0.959
4	6303668	6303668	G	A	*WFS1*	NM_006005.3	c.2146G>A	p.A716T	T	D	D	A	M	0.00001	0	AD_Pathogenic	3	0	1661	4740	0	4690	> 8.5	0.0	0.9 - 81.5	NA	0.024	NA
4	6303707	6303707	G	A	*WFS1*	NM_006005.3	c.2185G>A	p.D729N	T	B	D	D	L	0.00010	0	AD_Pathogenic	1	1	1663	4739	2	4738	1.4	0.5	0.1 - 15.7	0.0 - 5.5	0.713	1.000
4	6304029	6304029	A	C	*WFS1*	NM_006005.3	c.2507A>C	p.K836T	T	D	D	D	L	.	.	AD_Pathogenic	6	1	1658	4739	0	4690	> 17.0	> 1.0	2.0 - 141.1	0.1 - 15.8	0.000	0.996
4	6304030	6304030	G	C	*WFS1*	NM_006005.3	c.2508G>C	p.K836N	T	D	D	D	L	.	.	AD_Pathogenic	3	0	1661	4740	0	4690	> 8.5	0.0	0.9 - 81.5	NA	0.024	NA
4	6304112	6304112	G	A	*WFS1*	NM_006005.3	c.2590G>A	p.E864K	D	D	D	A	L	.	.	AD_Pathogenic	4	0	1660	4740	0	4690	> 11.3	0.0	1.3 - 101.2	NA	0.005	NA

Abbreviations: PP2: PolyPhen2, ExAC_all: Minor allele frequencies in the Exome Aggregation Consortium database, ExAC_eas: Minor allele frequencies in the East Asian population in the Exome Aggregation Consortium database, AD vs. CNT OR: Odds ratio of autosomal dominant or mitochondrial cases compared with JPN_CNT.AR vs. CNT OR: Odds ratio of autosomal recessive or sporadic cases compared with JPN_CNT. JPN_CNT alt: Total alternative allele number in Japanese control population including 2KJPN, HGVD, in house control. JPN_CNT ref: Total reference allele number in Japanese control population including 2KJPN, HGVD, in house control.

* Position was on the human genome version GRCh37/hg19.

# Supp. Table S2. Minor allele frequency and *in silico* prediction results of the variants described in the manuscript.

Case 1

Chr	Start	End	Ref	Alt	Variant	1000G ALL	1000G AFR	1000G AMR	1000G EAS	1000G EUR	1000G SAS	ExAC Freq	ExAC AFR	ExAC AMR	ExAC EAS	ExAC FIN	ExAC NFE	ExAC OTH	ExAC SAS	ESP6500si_ALL	ESP6500si_AA	ESP6500si_EA	SIFT score	SIFT pred	PP2 score	PP2 pred	LRT score	LRT pred	MutTaster score	MutTaster pred	MutAssessor score	MutAssessor pred	final conclusion
chr11	76867714	76867714	C	G	*MYO7A*:NM_000260.3:exon6:c.C479G:p.S160C	.	.	.	.	.	.	.	.	.	.	.	.	.	.	.	.	.	0	D	1	D	0	D	1	D	4.61	H	Likely Pathogenic
chr11	76893039	76893039	G	T	*MYO7A*:NM_000260.3:exon24:c.G2947T:p.D983Y	.	.	.	.	.	.	.	.	.	.	.	.	.	.	.	.	.	0.06	T	0.523	P	0	D	1	D	2.495	M	Uncertain significance

Case 2

Chr	Start	End	Ref	Alt	Variant	1000G ALL	1000G AFR	1000G AMR	1000G EAS	1000G EUR	1000G SAS	ExAC Freq	ExAC AFR	ExAC AMR	ExAC EAS	ExAC FIN	ExAC NFE	ExAC OTH	ExAC SAS	ESP6500si_ALL	ESP6500si_AA	ESP6500si_EA	SIFT score	SIFT pred	PP2 score	PP2 pred	LRT score	LRT pred	MutTaster score	MutTaster pred	MutAssessor score	MutAssessor pred	final conclusion
chr10	73499504	73499504	A	G	*CDH23*:NM_022124.5:exon34:c.4463A>G:p.E1488G	.	.	.	.	.	.	.	.	.	.	.	.	.	.	.	.	.	.	.	1	D	0	D	1	D	4.21	H	Likely Pathogenic
chr10	73560493	73560493	G	A	*CDH23*:NM_022124.5:exon51:c.7463G>A:p.R2488H	.	0.001	0.003	0.0014	.	.	.	0.0002	0.0019	0.0004	0.0001	0	6.10E-05	0	0	0.0008	0.0025	0.024	D	0.976	D	0	D	1	D	1.52	L	Likely Pathogenic
